# Sunflower and Watermelon Seeds and Their Hybrids with Pineapple Leaf Fibers as New Novel Thermal Insulation and Sound-Absorbing Materials

**DOI:** 10.3390/polym15224422

**Published:** 2023-11-16

**Authors:** Mohamed Ali, Zeyad Al-Suhaibani, Redhwan Almuzaiqer, Khaled Al-Salem, Abdullah Nuhait, Fahad Algubllan, Meshari Al-Howaish, Abdullah Aloraini, Ibrahim Alqahtani

**Affiliations:** Mechanical Engineering Department, College of Engineering, King Saud University, P.O. Box 800, Riyadh 11421, Saudi Arabiakalsalem@ksu.edu.sa (K.A.-S.);

**Keywords:** utilization of agrowaste materials, thermal insulation materials, sound absorption materials, thermal conductivity coefficient, pineapple leaf fiber, striped sunflower seed fiber, watermelon seed

## Abstract

Pineapple leaf fiber (PALF), striped sunflower seed fiber (SFSF), and watermelon seed (WMS) are considered natural waste polymer materials, which are biodegradable and sustainable. This study presents new novel thermal insulation and sound absorption materials using such waste as raw materials. PALF, SFSF, and WMS were used as loose, bound, and hybrid samples with different compositions to develop promising thermal insulation and sound-absorbing materials. Eleven sample boards were prepared: three were loose, three were bound, and five were hybrid between PALF with either SFSF or WMS. Wood adhesive was used as a binder for both the bound and hybrid sample boards. Laboratory scale sample boards of size 30 cm × 30 cm with variable thicknesses were prepared. The results show that the average thermal conductivity coefficient for the loose samples at the temperature range 20–80 °C is 0.04694 W/(m.K), 0.05611 W/(m.K), and 0.05976 W/m.K for PALF, SFSF, and WMS, respectively. Those for bound sample boards are 0.06344 W/(m.K), 0.07113 W/(m.K), and 0.08344 W/m.K for PALF, SFSF, and WMS, respectively. The hybrid ones between PALF and SFSF have 0.05921 W/m.K and 0.06845 W/(m.K) for two different compositions. The other hybrid between PALF and WMS has 0.06577 W/(m.K) and 0.07007 for two different compassions. The sound absorption coefficient for most of the bound and hybrid boards is above 0.5 and reaches higher values at some different frequencies. The thermogravimetric analysis for both SFSF and WMS shows that they are thermally stable up to 261 °C and 270 °C, respectively. The three-point bending moment test was also performed to test the mechanical properties of the bound and hybrid sample boards. It should be mentioned that using such waste materials as new sources of thermal insulation and sound absorption materials in buildings and other applications would lead the world to utilize the waste until zero agrowaste is reached, which will lower the environmental impact.

## 1. Introduction

Energy efficiency and sustainability have been a priority for almost all engineering applications for decades. Almost all fields of engineering are witnessing plans to cut down on energy consumption and increase sustainability. The European Union plans to reduce emission levels from the aviation and power industries by 21% compared to the 2005 emission levels, while for residential, transportation, and agriculture waste, this will be by about 20% compared to the 2005 emission levels [1]. Within the complex interaction between engineering developments and regulatory frameworks, the construction sector has seen a major increase in the adoption of environmentally sustainable materials [2,3,4]. In 2022, for similar reasons, the Kingdom of Saudi Arabia initiated a massive ecological campaign to plant 10 billion trees in the years to come. A growing number of countries are enacting environmental protection legislation, and this initiative is evidence of that trend [5]. Given the scale of this endeavor, effective waste management becomes imperative, not merely as a disposal concern but also as an avenue for resource optimization. One promising avenue for this waste is its integration into ecofriendly engineering solutions, particularly as a constituent material in sustainable construction practices. For example, solid waste, which is the byproduct of the pineapple processing industries, was estimated to be about 40–50% from fresh fruit as pineapple peelings, crowns, and cores [6]. Tangjuank [7] has reported the use of pineapple leaves as a good thermal insulation material. He developed insulation boards using natural rubber latex as a binder with different percentages. His results show a thermal conductivity coefficient range of 0.035 W/m.K to 0.043 W/m.K, which depends on the density of the board. Kumfu and Jintakosol [8] have produced thermal insulation boards using pineapple leaves with natural rubber latex as a binder at different percentages. Their developed boards had a thermal conductivity coefficient of 0.057 W/m.K at a density of 338 kg/m^3^ using a hot-pressing technique. Thilagavathi et al. [9] developed nonwovens from pure pineapple fibers and by blending polyester with pineapple fibers using the needle-punching technique. Their result showed that the nonwovens made by blending PALF with low-melt polyester had better sound and thermal insulation characteristics compared to pure PALF fibers. Do et al. [10] have produced flexible aerogel composites made from pineapple leaf and cotton waste fibers as thermal insulation materials. The thermal conductivity coefficient of their aerogel composites was in the range of 0.039–0.043 W/(m.K).

Striped sunflower seeds are primarily eaten as a snack food; as a result, they may be called confectionery sunflower seeds. When the sunflower seeds are dehulled, they produce a large number of wasted shells (hulls or husks). In 2018, the global production of sunflower seeds was 52 million tons, led by Ukraine with 27% and Russia with 25% of the world total. Argentina, Romania, and China also contributed significant volumes [11]. Sunflower stems, plaster, water, and sodium benzoate as a fungal inhibitor have been used to develop renewable composite boards for thermal insulation and sound absorption by Carvalho et al. [12]. Georgiev et al. [13] have shown that wheat straws and the husks of sunflower seeds can be used in a proper percentage to improve the physical and thermal properties of clay porous bricks. Sunflower stalks and cotton textile waste were used with an epoxy binder to form thermal insulation for buildings by Binici et al. [14]. The effect of moisture content on the thermal conductivity coefficient of sunflower seeds was studied by Darvishi and Zarein [15]. Their result showed a direct correlation between increasing the moisture content and decreasing the bulk density on the increased thermal conductivity. They obtained a thermal conductivity coefficient range of 0.1854 to 0.3047 W/(m.K). Binici et al. [16] have reported a compressed thermal insulation construction material made of vermiculite, wheat stalk, sunflower stalk, and gypsum as a binder. Their result showed a thermal conductivity coefficient of 0.063–0.334 W/(m.K) at a density of 0.166–0.302 g/cm^3^. This large number of wasted hulls will create a large environmental problem; they must be recycled in an economical and safe way.

Another source of waste is the hulls or shells of watermelon seeds. Statistics depict that the volume of watermelon produced in Saudi Arabia is approximately 216,708 tons [17]. Therefore, many seeds with or without kernels will produce an environmental problem and should be eliminated. On the other hand, Odewunmi et al. [18] have used the wasted watermelon seed extract as an effective corrosion inhibitor of mild steel in an HCl solution using electrochemical techniques at 25 °C.

There are studies [19,20,21,22] that have investigated the efficacy of several supplementary natural fibers as insulation materials. Plant materials encompass a diverse range of botanical resources, such as rice, date palm trees, wheat straw, kenaf, sugar cane, flax, and hemp. These plant materials exhibit considerable potential for utilization in numerous applications. Natural fibers are very promising and have great potential as ecofriendly raw materials to be used especially in insulation [23] and sound absorption [24,25]. Ali and Abdulkarem [26] have reported new thermal insulation materials extracted from date palm surface fibers. The thermal conductivity coefficient range of their produced sample boards was between 0.0475 and 0.0697 W/(m.K) using cornstarch resin as a binder. Fouladi et al. [27] have studied the sound absorption coefficients of four different fibers: coir, corn, sugar cane, and dry grass with different panel thicknesses and compared them with common building and acoustic panel materials, discovering that they are outstanding alternatives. Berardi and Iannace [28] have reported sound absorption and noise reduction coefficients for some natural fibers, such as wood, kenaf, hemp, cork, coconut, cane, sheep wool, and cardboard. Their results show that those coefficients depend on the thickness, porosity, and density of the materials, and they have suggested that those materials be used in buildings. 

The objective of this study is to evaluate the feasibility of utilizing fibers obtained from pineapple leaves, sunflower seeds, and watermelon seeds as raw natural polymer materials for thermal insulation and sound absorption. To the best of the authors’ knowledge, there is currently a lack of research that specifically investigates the utilization of these particular natural materials for the designated insulation purposes. The utilization of natural wood adhesive in the assembly of these components provides additional support for the adherence of the study to the principles of environmental sustainability. Therefore, this study is a novel addition to the current collection of knowledge on environmentally conscious construction methods and sustainable building principles. 

## 2. Materials and Methods

### 2.1. Obtaining and Preparing the Raw Materials

**Figure 1:**
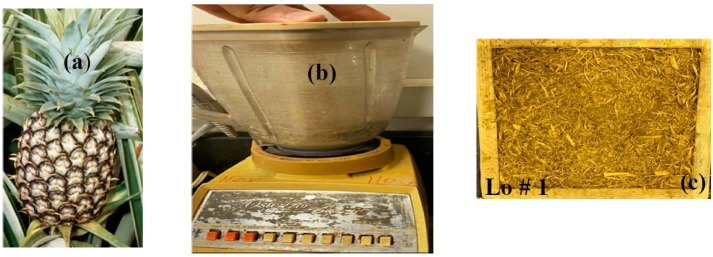
Preparation of pineapple leaf fiber; (**a**) crown leaves, (**b**) blender used for grinding the crown leaves, and (**c**) pineapple leaf fiber (PALF) (loose sample # 1).

Three waste materials were used in this study, namely, pineapple leaf fiber (PALF), sunflower seed fiber (SFSF), and watermelon seed (WMS). The pineapple leaves were extracted and collected from the crown leaves of the pineapple fruit, washed to remove any dust or impurities, and dried in the sun at 40 °C for three days and then moved to an electric oven for a few hours at 100 °C until they were completely dried with no condensed water vapor on the glass of the oven. The dried hot samples were left in the laboratory until they absorbed the moisture of the laboratory (RH = 51.7) at room temperature of 21.6 °C. A blinder was used to grind the leaves into fibers as shown in Figure 1. The pineapple leaves were usually collected from the local juice stores as daily waste. The ground leaves had an average approximate length of 1–3 cm as shown in Figure 1c. It should be noted that the ground pieces (Figure 1c) were used as they were without any kind of chemical treatment. 

**Figure 2:**
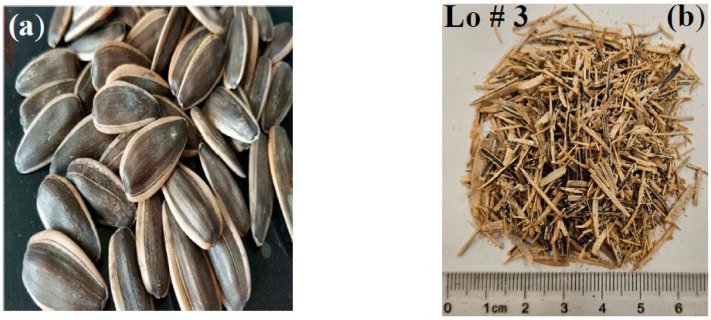
Extraction of sunflower seed hulls; (**a**) sunflower seeds and (**b**) ground hulls (loose sample # 3).

**Figure 3:**
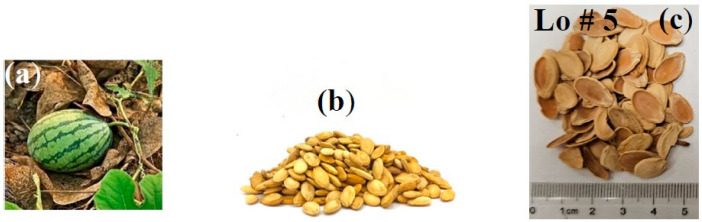
Extraction of watermelon seeds; (**a**) watermelon, (**b**) extracted seeds, and (**c**) seed shells (loose sample # 5).

Sunflower seed hulls are an abundant waste produced by the edible oil industry. Therefore, the objective was to use those hulls to produce new insulation materials by testing them as loose or bound hulls. Sunflower seed hulls were collected from some well-known companies in our local area. The collected hulls were washed, dried, and ground similar to the PALF mentioned earlier. Figure 2 shows the sunflower seeds and the ground hulls. Watermelon (Citrullus lanatus) is consumed all over the world and contains a large number of seeds, which are discarded. Properly using those seeds will solve some of the environmental problems. The watermelon seeds were collected as they were, washed, and dried as mentioned earlier. Figure 3 shows the watermelon and the extracted seeds.

### 2.2. Preparing the Samples for Testing

**Figure 4:**
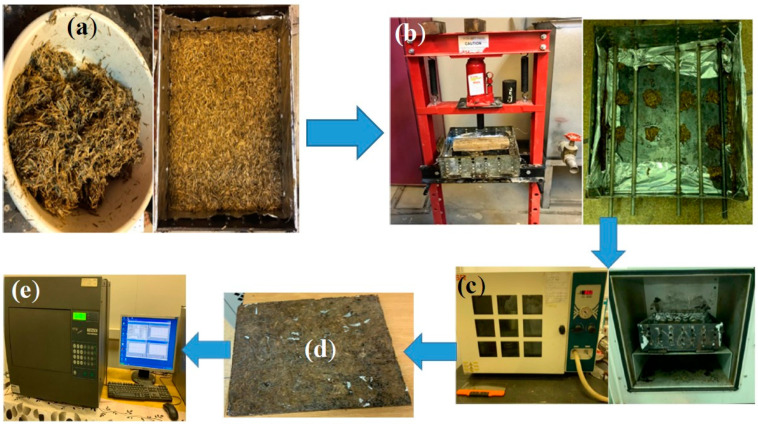
Preparing the samples for thermal conductivity measurements. (**a**) mixing materials with the binder in the mold, (**b**) pressing the material, (**c**) drying oven, (**d**) extracting drying sample, and (**e**) heat flow meter for thermal conductivity measurements.

Different molds were prepared to hold the different samples either as loose materials, bound, or as a hybrid using wood adhesive as a binder (Table 1 shows the characteristics of the adhesive). Figure 1c above shows the wood mold used to hold the loose materials with inside dimensions of 26.5 × 26.5 × 2.0 cm^3^ to be suitable for thermal conductivity measurements as will be shown later. Different bound or hybrid material samples were prepared, mixed with the wood adhesive solution with specific concentration, put in the mold, and moved to the presser and then to the oven for drying followed by the heat flow meter for thermal conductivity measurements. The mold dimensions are 30 × 30 × H cm^3^, where H presents the variable height (thickness) up to 10 cm. Figure 4 shows the preparation steps used in producing the bound or hybrid samples on the laboratory scale.

**Figure 5:**
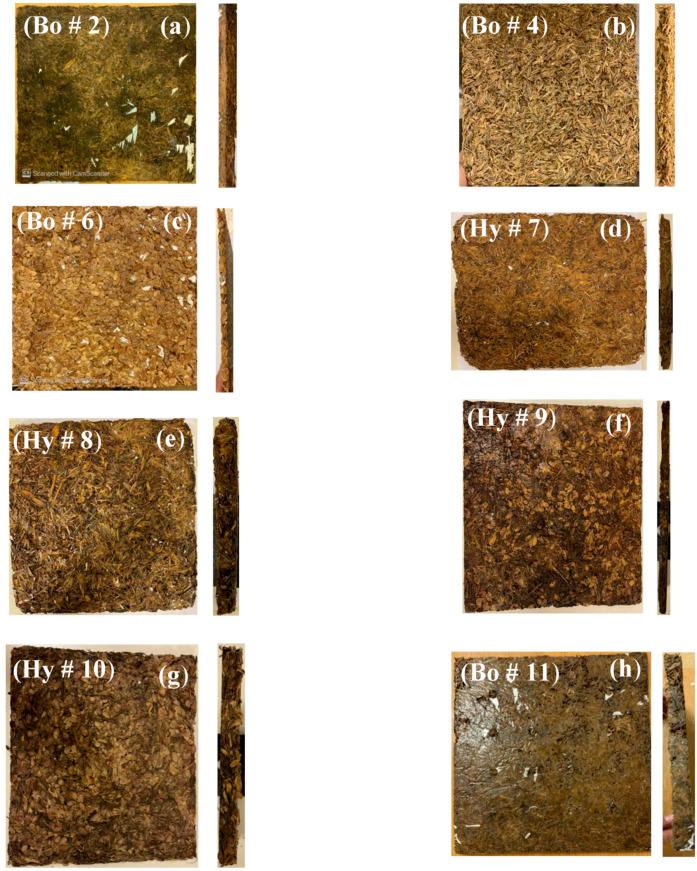
Laboratory-prepared samples with size 30 × 30 cm^2^ and with different thicknesses (**a**) bound PALF sample # 2, (**b**) bound SFSF sample # 4, (**c**) bound WMS sample # 6, (**d**) hybrid of PALF and SFSF sample # 7, (**e**) hybrid of PALF and SFSF sample # 8, (**f**) hybrid of PALF and WMS sample # 9, (**g**) hybrid of PALF and WMS sample # 10, and (**h**) sunflower seed fiber (SFSF) bound sample with high binder ratio # 11 (see Table 2 for more details).

Table 2 shows the specifications, dimensions, density, binder ratio, and others for the produced samples, and Figure 5 shows the laboratory-prepared bound and hybrid samples of size 30 × 30 cm^2^ with different thicknesses as listed in Table 2.

## 3. Laboratory Tests

### 3.1. Three-Point Bending Moment Test

**Figure 6:**
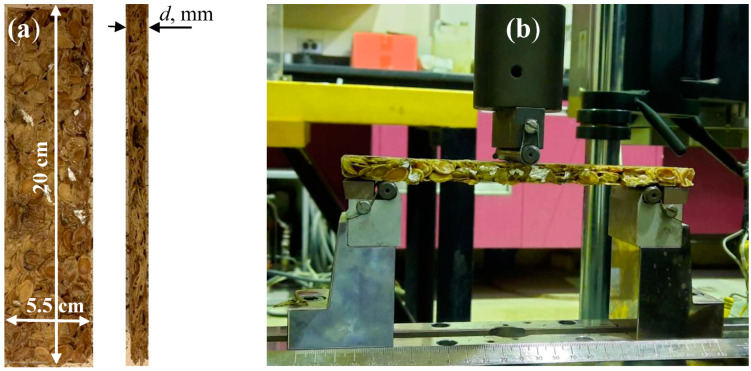
Bending specimen dimensions (**a**) and (**b**) the universal testing machine (UTM, INSTRON 5984) used to perform the bending moment test.

The three-point bending moment test was obtained for all bound and hybrid composite samples listed in Table 2. The test was performed on the cut specimens shown in Figure 6a. The dimensions of the specimens are 20 cm × 5.5 cm × *d*, where *d* is a variable thickness corresponding to each specimen as shown in Table 2. The important bending parameters, such as bending force *F* (N), deflection *D* (mm), flexural stress *σ_f_*, and flexural strain ϵ*_f_*, were obtained for each specimen using the universal testing machine (UTM, INSTRON 5984) (Figure 6b) of crosshead speed of 2 mm/min. The definition of *σ_f_*, *ε_f_*, and flexural elastic modulus *E_f_* is defined by

(1) $$\sigma_{f}=\frac{3FL}{2bd^{2}},\;\epsilon_{f}=\frac{6Dd}{L^{2}},\;E_{f}=\frac{L^{3}S}{4bd^{3}}$$

All other parameters are defined in Table 3. This test followed the standard ASTM D790-03 [33].

### 3.2. Thermal Stability Test

Thermal stability test analyses were characterized for both the raw materials of sunflower (SF) and watermelon (WM) seeds using the thermogravimetric analysis (TGA), differential (DTGA), and differential scanning calorimetry (DSC). TA instruments (New Castle, DE, USA), SDT Q600 V20.9 Build 20 setup, which was fitted with a Nitrogen purge gas, was used to conduct the thermogravimetric analysis test. Initially, 7.27 mg and 6.57 mg were used for SFS and WMS, respectively. Each amount was put in an alumina pan and heated up to 550 °C. The initial temperature, heating rate, and mass flow rate of the Nitrogen gas are 25 °C, 10 °C/min, and 100 mL/min, respectively. The TGA and the DSC test analyses followed ASTM E1131-08 [34] and ASTM D3418 [35] standards, respectively.

### 3.3. Thermal Conductivity Test

Thermal conductivity coefficients were determined at a temperature range of about 20 °C to 80 °C for all samples shown in Table 2 and Figure 5. Lambda heat flow meter (HFM, 436), bench type (Figure 4e), was used for thermal conductivity coefficient determination. The method of measurement followed the guarded hot plate standard method ASTM-C518 [36]. The HFM can enclose any sample of specific size 30 cm × 30 cm × H, where the thickness H can vary up to 10 cm. The created heat flow between the hot and cold plates was at a 20 °C mean temperature difference. The HFM has a self-automated sensor to measure the sample’s thickness in cm. The accuracy of measuring the temperature and the thermal conductivity coefficient is ±0.01 °C and ±1% to 3% W/mK, respectively, as provided by the manufacturer.

### 3.4. Sound Absorption Test

**Figure 7:**
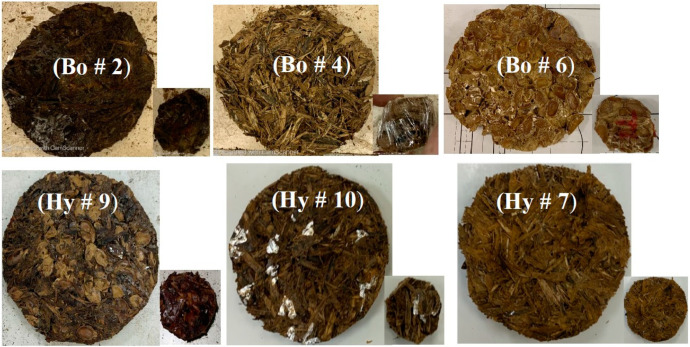
Specimens used for sound absorption coefficient for 3 and 10 cm in diameter; their thickness are given in Table 2.

BSWA Technology Ltd.’s (Beijing, China) impedance tube with different diameters was used to determine the sound absorption coefficients (SACs) at different frequencies. Specimen sizes of 3 and 10 cm in diameter were prepared from the bound (Bo) and hybrid (Hy) samples for SAC measurement as shown in Figure 7. Complete details and descriptions of the BSWA tubes, microphones, and principles of operation can be obtained from our previous publication, Ali et al. [37]. The impedance tube test procedure followed ISO 10534-1 [38] and ISO 10534-2 [39] standards. 

### 3.5. Moisture Content Test

**Figure 8:**
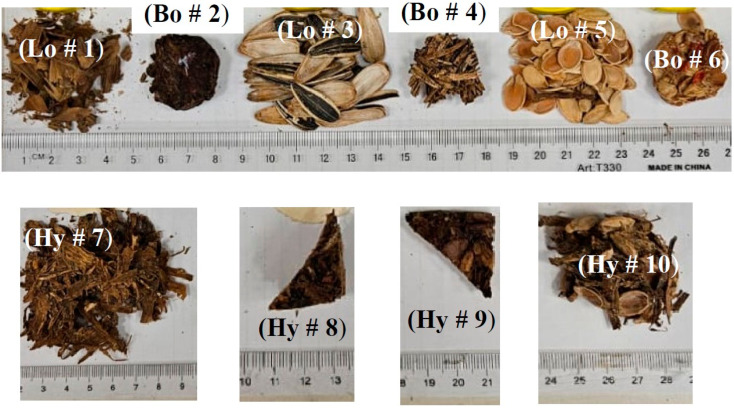
Small pieces used for the moisture content test.

Small amounts (a few grams, Figure 8) of the loose, bound, and hybrid samples were dried in the convection oven (Figure 4c) for 24 h; then their masses were recorded as the dried masses and named *m*2. After that, their masses were triggered every 5 min (*m*1) in the laboratory environment with a temperature and relative humidity of 21.6 °C and 51.7%, respectively, until they reached a steady state of constant values. The percentage amount of absorbed moisture was calculated using Equation (2), which followed the ASTM D2974-07A [40] standard.

(2) $$\%\;\mathrm{of}\;\mathrm{moisture}\;\mathrm{content}=\frac{m1-m2}{m2}\times 100$$

## 4. Results and Discussion

The results are presented for all tests conducted and described in Section 3; the sample specifications are shown in Table 2 and Figure 5, Figure 6, Figure 7 and Figure 8.

### 4.1. Bending Moment Test

**Figure 9:**
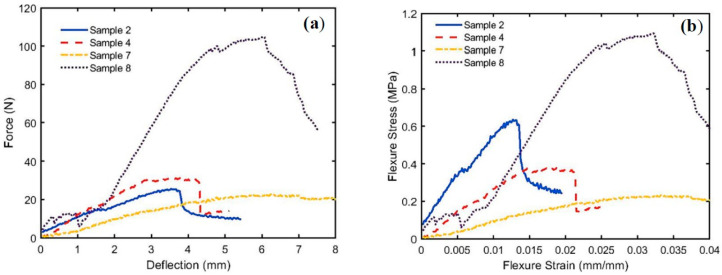
Bending parameters for bound and hybrid specimens of PALF and SFSF; (**a**) load-deflection and (**b**) stress–strain curves.

Figure 9a,b show the load-deflection and flexural stress $\sigma_{f}$ profiles, respectively, for the bound specimens of PALF (Bo # 2), SFSF (Bo # 4), a hybrid of both (Hy # 7), and (Hy # 8). The calculated parameters, such as $\sigma_{f}$, ϵ*_f_*, $E_{f}$, and the slope S, are shown in Table 4 for each specimen.

**Figure 10:**
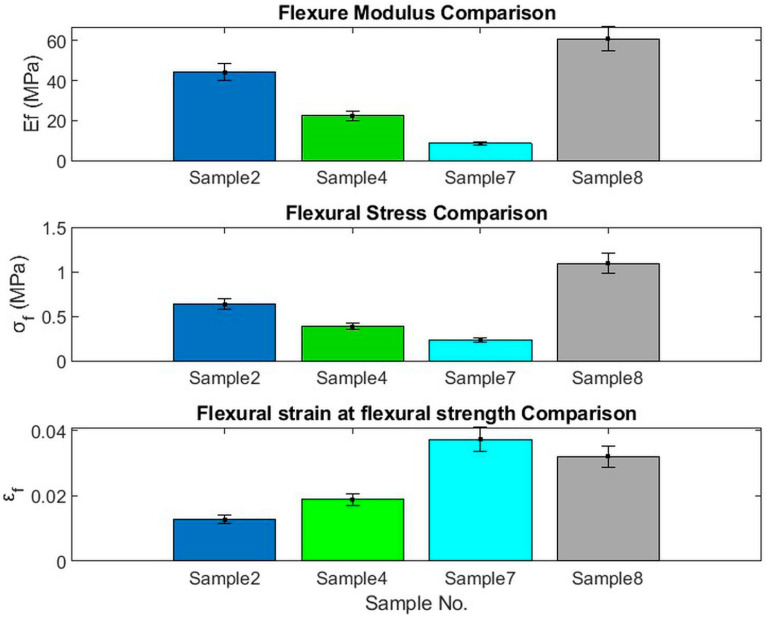
Comparison among $E_{f}$, $\sigma_{f}$, and *ϵ_f_* for specimen numbers 2, 4, 7, and 8 for bound and hybrid between PALF and SNSF.

It should be noted that the slope *S* was calculated for the initial straight-line profiles shown in Figure 9a up to the elastic limit. The flexural strain at flexural strength $\epsilon_{f}$ was obtained from the stress–strain curves (Figure 9a) when the curves start to deviate from linearity [41]. Figure 10 shows a comparison between those parameters. It should be noted that the flexural modulus $E_{f}$, flexural stress $\sigma_{f}$, and *ϵ_f_* depend on the degree of compactness, the polymerized binder ratio, and the thickness of the specimen. Consequently, specimen numbers 2 and 8 have a higher density (more compact) than the others do; therefore, they reflect higher $E_{f}$ and $\sigma_{f}$ values, and, in addition to that, the percentage of polymerized binder for # 8 is higher than that of # 2.

**Figure 11:**
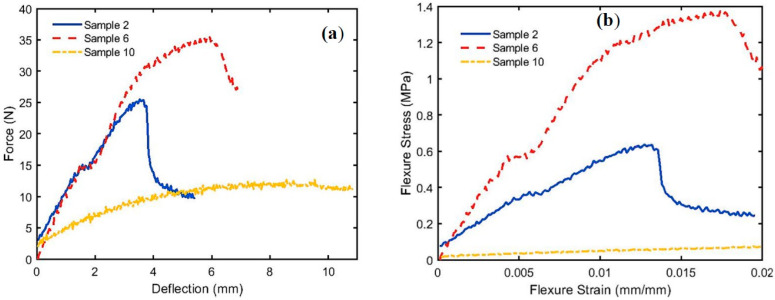
Bending parameters for bound and hybrid specimens of PALF and WMS; (**a**) load-deflection and (**b**) stress–strain curves.

**Figure 12:**
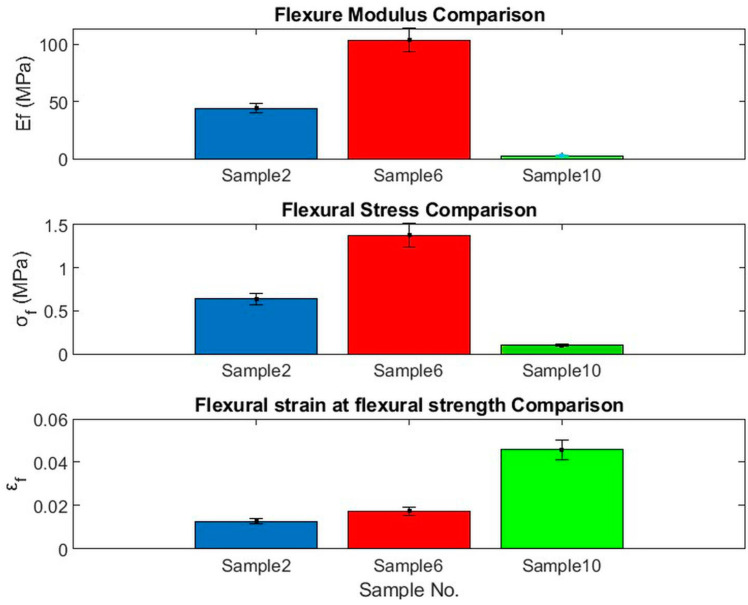
Comparison among $E_{f}$, $\sigma_{f}$, and *ϵ_f_* for specimen numbers 2, 6, and 10 for bound and hybrid between PALF and WMS.

Figure 11 shows the load-deflection and flexural stress $\sigma_{f}$ profiles, respectively, for the bound specimens of PALF (Bo # 2), WMS (Bo # 6), and hybrid (Hy # 10). Figure 12 shows a comparison between the bound (# 2, and 6) and hybrid (# 10) specimens of PALF and WMS. The same trends were observed as shown in Figure 9 and Figure 10 for the bound and hybrid specimens of PALF and SFSF. The numerical values of governing parameters, such as $E_{f}$, $\sigma_{f}$, and *ϵ_f_*, are shown in Table 4, and the specimen dimensions are shown in Table 3, which is mentioned earlier. Figure 12 indicates that specimen number 6 has better performance because it has a higher density (more compact) and has a high percentage of polymerized binder (31%). The percentage of uncertainty was calculated following the procedure described by Moffat [42], and a computer program was written to perform that. The maximum uncertainties of Flexural Modulus (*E_f_*), Flexural Stress (*σ_f_*), and Flexural Strain (*ε_f_*) are 8.23%, 5.50%, and 2.76%, respectively. The error bars are added in Figure 10 and Figure 12 for all samples.

### 4.2. Thermal Stability Test Analyses

**Figure 13:**
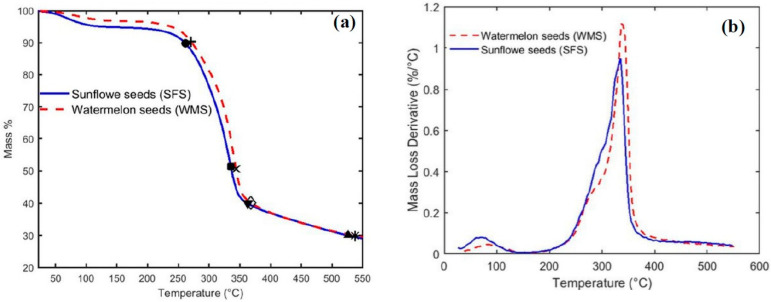
Thermogravimetric analyses for both sunflower and brown seeds; (**a**) TGA profiles and (**b**) derivative thermogravimetric analyses (DTGAs).

Comparisons between the thermogravimetric analysis (TGA) profiles of sunflower (SF) and watermelon (WM) seeds are shown in Figure 13a, and their derivative thermogravimetric analyses (DTGAs) are constructed in Figure 13b. Figure 13a shows that the raw materials of both seeds are thermally stable up to about 261 °C (●) and 270 °C (+) for both SFS and WMS, respectively, where they only lost 10% of their mass. The major loss of mass for SFS is between 261 °C (●) and 363 °C (▼), where the material lost 60% of its mass. On the other hand, WMS lost 60% of its mass between 270 °C (+) and 368 °C (◊). Both seeds almost reached a char at 526 °C (▲) and 538 °C (*) for SFS and WMS, respectively, where they lost almost 70% of their masses. Both SFS and WMS decompose to about 50% of their mass at 335 °C (■) and 440 °C (×), respectively. The degradation of the temperature range and its peak for both SFS and WMS is shown in Figure 13b. Consequently, Figure 13a,b ensure that both raw materials of SFS and WMS are thermally stable at high temperatures. Therefore, these materials with their high thermally stable temperatures can be used as new novel thermal insulation materials for buildings and could enhance our environmental future by using such new natural and biodegradable materials that are safe for humans to replace both synthetically and petrochemically produced thermal insulation materials. 

**Figure 14:**
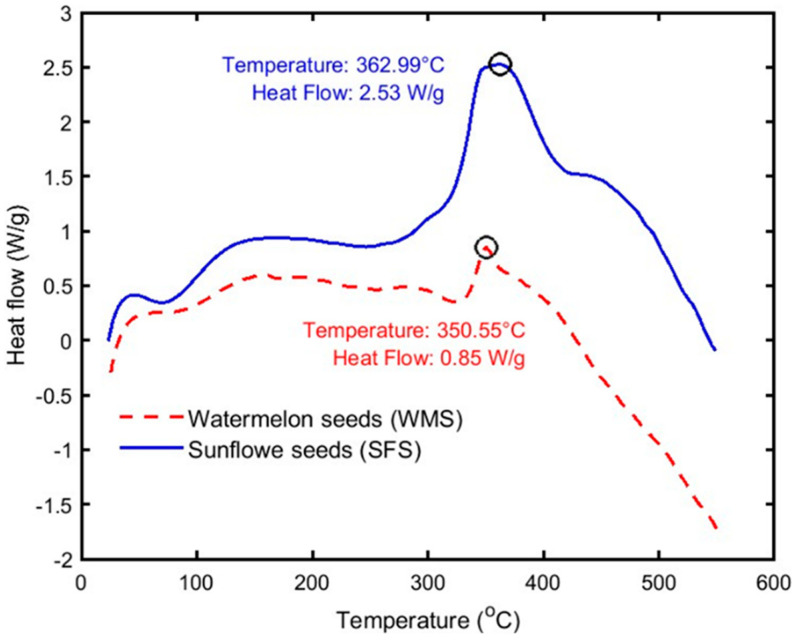
The Differential Scanning Calorimetry (DSC) analysis for both the sunflower (SF) and the watermelon (WM) seeds.

Figure 14 shows that the endothermic transition starts at about 363 °C and 351 °C for SFS and WMS, respectively, and they both reach a maximum at 550 °C. At these two temperatures, the samples lost about 60% and 55% of their mass as indicated by the TGA analysis shown in Figure 13a. Therefore, these two endothermic transition peaks could be defined as the initial melting points of these two materials. Consequently, The DSC analysis indicates that these two fibers are stable up to 261 °C and 270 °C for SFS and WMS as also indicated by Figure 13a, where they almost lost only the moisture content. 

### 4.3. Thermal Conductivity Coefficient Analyses

**Figure 15:**
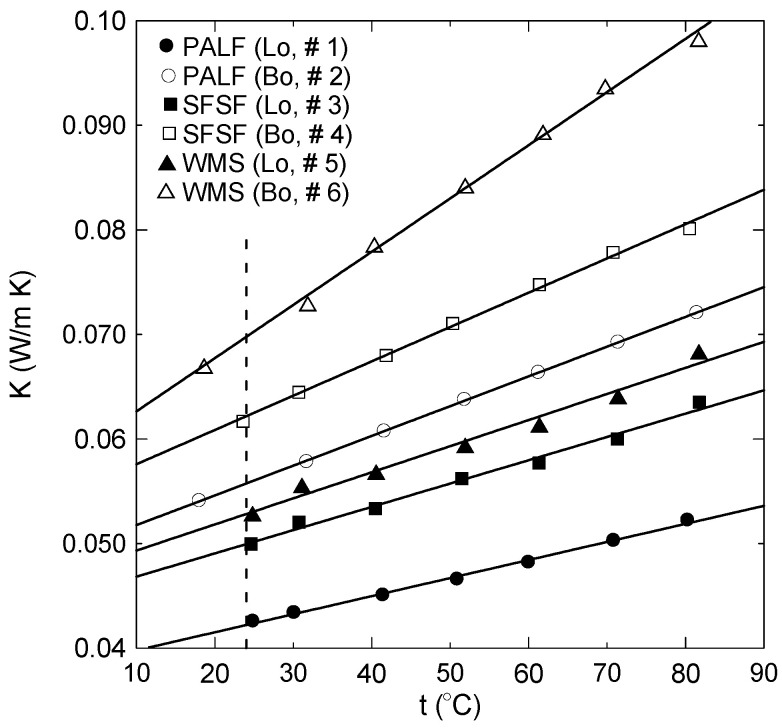
Thermal conductivity coefficients for loose and bound samples of PALF, SFSF, and WMS; solid lines present the curve fitting through the data.

Figure 15 shows the thermal conductivity coefficients for loose and bound samples of PALF (● #1, ○ #2), SFSF (■ #3, □ #4), and WMS (▲ #5, Δ #6). As indicated, all the loose samples have lower thermal conductivity coefficients (K) than the bound samples. This could be attributed to the air gaps between the loose fibers, which have low thermal conductivity. However, most of those air gaps were filled by the used binder, which has higher thermal conductivity. Therefore, the result is an increase in the effective thermal conductivity coefficients of the bound samples compared to the loose ones. The vertical dashed line shows that the thermal conductivity coefficients of all samples are less than 0.07 W/m.K at the ambient temperature of 24 °C, which allows them to be used for thermal insulation. All samples have linear correlation to cover a temperature range greater than 20 °C and less than 80 °C in the form:

K = A + B × t(3)

**Figure 16:**
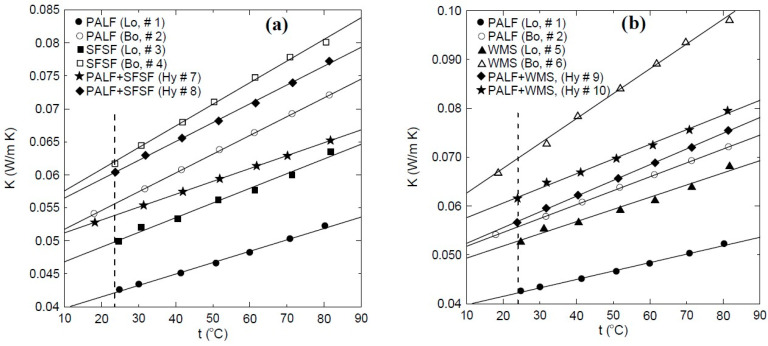
Comparison between the thermal conductivity coefficients for loose, bound, and hybrid samples; solid lines present the curve fitting through the data. (**a**) PALF and SFSF and (**b**) PALF and WMS.

**Figure 17:**
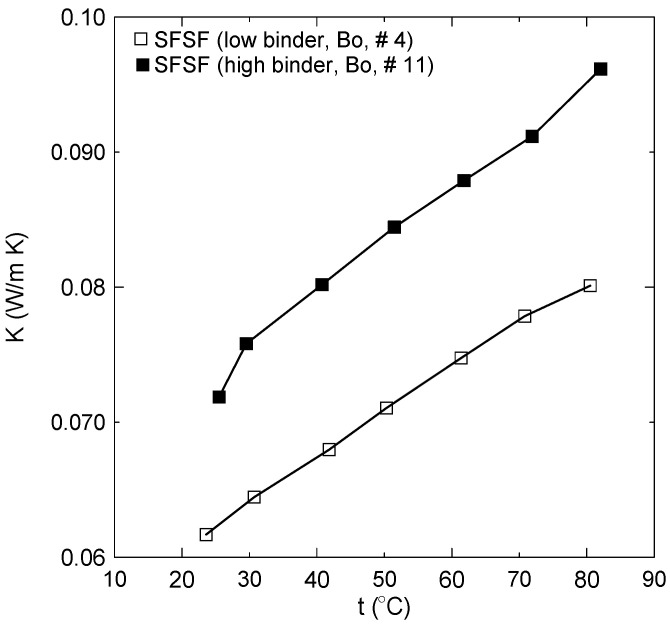
The effect of the binder ratio on the thermal conductivity coefficient profiles.

**Figure 18:**
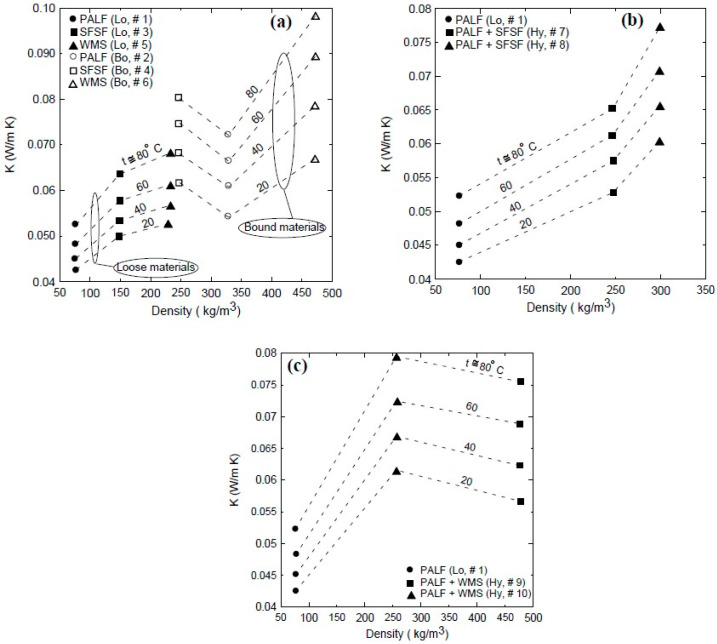
Thermal conductivity coefficient profiles at different temperatures versus the density of the samples. (**a**) Loose and bound single-material samples. (**b**) Hybrid samples of PALF and SFSF. (**c**) Hybrid samples of PALF and WMS.

Table 5 shows the constants A and B and the coefficient of determination for each linear regression. Figure 15 shows also that the thermal conductivity coefficient is almost a linear function of temperature and increases in the temperature range of 20 °C–80 °C as 22.7%, 33.2%, 27.1%, 29.8%, 29.3%, 46.7%, 23.5%, 27.9%, 33.2%, and 29.1% for sample numbers 1, 2, 3, 4, 5, 6, 7, 8, 9, and 10, respectively, as shown in Table 2. Figure 16a shows a comparison between the thermal conductivity coefficient profiles of the bound samples of PALF and SFSF and their hybrid samples 7 and 8. It is clear that as the percent of PALF increases in the hybrid sample (#7), this leads to an enhancement in the thermal conductivity coefficient compared to that of the bound sample (#4) of SFSF, and the opposite is true as shown in sample number 8. Figure 16b shows similar profiles of the thermal conductivity coefficients for the bound and hybrid samples except for PALF and WMS. The same enhancement trend in the thermal conductivity coefficient is observed as the percent of PALF is increased in the hybrid samples as shown in sample # 9 compared to sample number 10. The vertical dashed line shown in Figure 16 indicates that the thermal conductivity coefficients of all samples are less than 0.07 W/m.K at the ambient temperature, which allows them to be used as thermal insulation materials for buildings. It should be noted that increasing the binder ratio in the bound or hybrid sample would deteriorate the thermal conductivity coefficient. Therefore, Figure 17 is constructed to show this effect, where sample number 11 (■) has a polymerized mass percent of 54 compared to number 4 (**□**) of 31 using SFSF as the material. The increase in the thermal conductivity coefficient is about 19% at a temperature 50 °C, where the mass of the binder increases by about 159%. Table 6 shows a comparison between the current finding of the thermal conductivity coefficients and those of some similar natural thermal insulation materials available in the literature. Figure 18a–c show the thermal conductivity coefficient profiles at different temperatures versus the density of the samples shown in Table 2. Figure 18a presents two different sets of profiles, one for loose materials (solid symbols) of PALF, SFSF, and WMS and the other (hollow symbols) for bound ones. This figure indicates that the thermal conductivity coefficients in general increase as either the temperature or the density increases for a fixed volume of the material as shown for the loose samples. However, the thermal conductivity coefficients still increase for the bound samples compared to their corresponding loose ones. It should be noted that each symbol presents a different sample along each isothermal profile as shown in the legend of the figure. Figure 18b,c show the same profiles but for hybrid samples of (PALF, SFSF) and (PALF, WMS), respectively. It is worth mentioning that the second and third symbols along each isothermal line present the hybrid samples compared to the first sample of the loose materials. Figure 18b,c indicate that the thermal conductivity coefficients are a function of both the temperature and the density and they increase as each of them increases compared to that of the loose material.

### 4.4. Sound Absorption Coefficient Determinations

**Figure 19:**
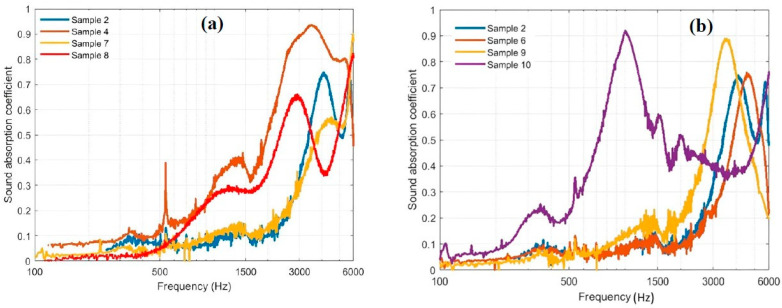
Sound absorption coefficient profiles for (**a**) bound and hybrid samples of PALF and SFSF and (**b**) bound and hybrid samples of PALF and WMS.

Sound absorption coefficients (SACs) were measured for samples 2, 4, 6, 7, 8, 9, and 10 covering the whole frequency range up to 6000 Hz using the impedance tubes of 3 and 10 cm diameters as described in Section 3.4. Samples 2, 4, and 6 are for Bound PALF, SFSF, and WMS. Samples 7, 8, 9, and 10 are for the hybrid ones. Figure 19a presents the SACs for samples 2, 4, 7, and 8, which involve bound PALF and SFSF and their hybrids. The SAC for sample 2 is greater than 0.5 for a frequency greater than 3500 Hz with two bell shaped maximums at 4000 Hz and 5600 Hz, where the SACs are 0.73 and 0.66, respectively. Sample number 4 has a better SAC above 0.8 for a frequency range of 2500 to 5500 Hz. Sample number 7 has an SAC greater than 0.5 for a frequency greater than 4000 Hz and reaches 0.9 at 6000 Hz. Sample number 8 has an SAC greater than 0.5 for a frequency range of 2500 Hz to 3500 Hz and values greater than 5000 Hz. Figure 19b shows the profiles for the SACs for samples 2, 6, 9, and 10, which present the bound samples of PALF and WMS and their hybrids, respectively. Sample number 6 has an SAC greater than 0.5 for a frequency range of 3800 Hz to 5500 Hz with a peak of 0.74 at 4750 Hz. Sample 9 has a better SAC greater than 0.5 at a frequency range of about 2800 Hz to 4800 Hz with a peak of ≈0.9 at about 3500 Hz. On the other hand, sample number 10 has a good SAC at a low frequency range of about 750 Hz to 1500 Hz with a peak of about 0.9 at 1000 Hz. This figure ensures that the bound sample # 4 gives a better SAC for a wider frequency range than the hybrid ones as shown in Figure 19a. On the other hand, hybrid sample # 9 presents a better SAC than the other bound samples as shown in Figure 19b. In general, most of the samples show a good ability to absorb the sound waves and therefore could be used as sound absorption materials for buildings and other applications. It should be noted that the presence of air passages in the samples help absorb an echo, which in turn gives a higher SAC. This could be attributed to the fact that some samples have a better SAC than others, which is based on the structure rigidity of the sample, which in turn depends on the amount of polymerized binder in the sample. Table 7 shows the noise reduction (NRC) coefficient, which was calculated by using the mean value from four 1/3 octave values of the SAC (250 Hz, 500 Hz, 1000 Hz, and 2000 Hz). The result is rounded to the nearest 0.05 [28,46]. Table 7 also compares the NRC of the current boards with some similar boards and fibers in the literature using the same technique. It should be mentioned that the NRC depends on the material type, density, thickness, and porosity. It is noted that the NRC decreases as the density of the board increases, which means more reflection than absorption [28,47].

### 4.5. Moisture Content

**Figure 20:**
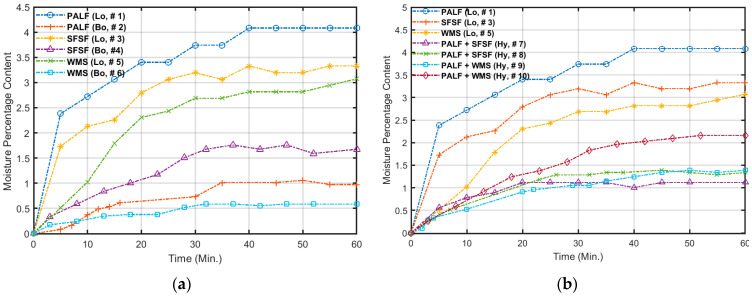
Percentage of moisture content in (**a**) loose and bound samples and (**b**) loose and hybrid samples.

The moisture content profiles for the loose and bound samples as a group are shown in Figure 20a,b for loose and hybrid samples as another group. The test was performed as described in Section 3.5 at the laboratory conditions of t = 21.6 °C, RH = 51.7. Figure 20a indicates that the maximum moisture content was observed by the loose samples followed by the bound samples. The moisture content for the loose samples is in the range of 3.1–4.1% and that of the bound samples is in the range of 0.6–1.7%. On the other hand, the moisture content for the hybrid samples is in the range of 1.1–2.2% as shown in Figure 20b. It is clear that as the samples become bound or hybrid, their ability to absorb water decreases because most of the pores (voids and spaces) are already filled by the polymerized binder. These low percentages of moisture content allow these new materials to be used as thermal insulation materials because the thermal conductivity coefficient is moisture-dependent and deteriorates as the moisture content increases. It should be noted that Bainbridge [50] has shown for a similar natural fiber (straw bale) that a 16% moisture content is safe.

## 5. Conclusions

New novel thermal insulation and sound absorption materials were developed and tested using the wasted pineapple leaf fiber (PALF), striped sunflower seed fiber (SFSF), and watermelon seed (WMS) as the raw materials. The new loose PALF, SFSF, and WMS have thermal conductivity coefficients of 0.04256, 0.04995, and 0.05284 W/m.K at an ambient temperature of about 24 °C. We learned that adding PALF to both SFSF and WMS in a large hybrid composition enhances the thermal conductivity coefficient more than the bound ones. The SFSF and the WMS are thermally stable at higher temperatures of 261 °C and 270 °C. Most hybrid and bound samples show a high sound absorption coefficient greater than 0.5 at a high frequency. However, sample # 10 shows a good acoustic characteristic at a low-frequency range of 750–1500 Hz. The bending moment tests for the bound and hybrid samples indicate that most of them have good mechanical characteristics. These conclusions ensure that the newly developed materials have a promising effect to be used as thermal and sound absorption materials for building and other applications and replace the thermal insulation materials produced from synthetic and petrochemicals. This will have a good environmental impact by removing huge amounts of waste while producing new natural, biodegradable, and ecofriendly thermal insulation and sound absorption materials.

## Figures and Tables

**Table 1:** Technical data of wood adhesive (78–1040) as provided by the manufacturer [29].

Ingredients			
Name	Case No.		Content %
Polyvinyl Acetate	9003-20-7		77–85
Water	1132-18-58		5–10
Dibutyl phthalate (DBP)	84-74-2		1–3
Calcium Carbonate	1317-65-3		10–20
Physical and chemical properties			
Base material	Polyvinyl Acetate	Coverage (Approx.)	4–5 m^3^/kg, depending on the surfaces
Color	Milky white	Application temperature	5–50 °C
Viscosity at 25 °C, ASTM D2196 [30]	25,000–34,000 CPS (Sp.# 7 at 20 rpm)	Drying time	30 min to approx. 1 h (depends on the thickness of the adhesive layer, kind of wood, and climatic conditions)
Density	1.10–1.5 g/cm^3^	Open time	1–5 min (depends on climatic conditions)
PH, ASTM D1172 [31]	6.5–8	Full cure	24 h (depending on climatic conditions)
Solid contents, ASTM D1644 [32]	51–56%	Pressing time	1–2 h (depending on climatic conditions and wood type)

**Table 2:** Matrix of the loose (Lo), bound (Bo), and hybrid (Hy) samples used in this study (PALF, SFSF, and WMS stand for pineapple leaf fiber, sunflower seed fiber, and watermelon seed, respectively). Wood adhesive was used as a binder for bound and hybrid samples.

Material	Sample Number										
	Lo (# 1)	Bo (# 2)	Lo (# 3)	Bo (# 4)	Lo (# 5)	Bo (# 6)	Hy (# 7)	Hy (# 8)	Hy (# 9)	Hy (# 10)	Bo (# 11)
PALF %	100	87	0.0	0.0	0.0	0.0	42.0	17.0	33.0	17.0	0.0
SFSF %	0.0	0.0	100	69	0.0	0.0	42.0	55.0	0.0	0.0	46.0
WMS%	0.0	00	0.0	0.0	100	69.0	0.0	0.0	33.0	52.0	0.0
The ratio of the polymerized binder to the total mass %	0.0	13	0.0	31	0.0	31.0	16.0	28.0	34.0	31.0	54.0
Thickness, H (mm)	21.0	13.0	21.0	21.0	21.0	11.0	21.0	22.0	11.0	26.0	18.0
Figure #	Figure 1c	Figure 5a	Figure 2b	Figure 5b	Figure 3b	Figure 5c	Figure 5d	Figure 5e	Figure 5f	Figure 5g	Figure 5h
Density of dried specimen (kg/m^3^)	76.5	329	148	248	235	472	247	299	478	256	432
Total dried mass (g)	106	370	210	469	333	467	475	592	486	600	700

**Table 3:** Dimensions and definitions of the parameters used in Equation (1).

Sample No.	Thickness *d*, (mm)	Width *b*, (mm)	Span, *L* (mm)	Slope (S) (N/mm)
2	13.0	49.5	150	6.39
4	21.0	54.0	150	9.20
6	11.0	48.0	150	7.85
7	21.0	52.0	150	4.51
8	22.0	49.0	150	31.13
10	26.0	48.0	150	1.98

**Table 4:** Important parameters for the three-point bending test for the specimens listed in Table 3.

Sample No.	Slope (N/mm), *S*	Flexure Modulus (MPa), *E_f_*	Flexural Stress (MPa), *σ_f_*	Flexural Strain at Flexural Strength, *ϵ_f_*
2	6.39	44.30	0.64	0.013
4	9.20	22.33	0.38	0.019
6	7.85	103.65	1.37	0.017
7	4.51	8.65	0.23	0.037
8	31.13	60.81	1.09	0.032
10	1.98	2.54	0.10	0.046

**Table 5:** Coefficients for linear regressions for all samples.

	Figure #	A	B	R^2^, %
Sample 1, ●	Figure 15	0.038	0.0002	99.6
Sample 2, ○	Figure 15	0.049	0.0003	99.9
Sample 3, ■	Figure 15	0.045	0.0002	98.9
Sample 4, □	Figure 15	0.054	0.0003	99.7
Sample 5, ▲	Figure 15	0.047	0.0002	98.0
Sample 6, Δ	Figure 15	0.058	0.0005	99.7
Sample 7, *	Figure 16a	0.049	0.0002	100.0
Sample 8, ♦	Figure 16a	0.054	0.0003	99.8
Sample 9, ♦	Figure 16b	0.049	0.0003	99.95
Sample 10, ⁎	Figure 16b	0.055	0.0003	99.6

**Table 6:** Comparison of the average thermal conductivity values with those in the literature for some unconventional insulation materials.

Natural Raw Materials	Density (kg/m^3^)	Thermal Conductivity (W/(m.K)	References
Bo PALF (#2)	329	0.0541–0.0721	Current
Bo SFSF (#4)	248	0.0617–0.0801	Current
Bo WMS (#6)	472	0.0669–0.0982	Current
Hy (#7)	247	0.0528–0.0652	Current
Hy (#8)	299	0.0604–0.0772	Current
Hy (#9)	478	0.0566–0.0755	Current
Hy (#10)	256	0.0616–0.0795	Current
Bound Eucalyptus globulus leaves	153.0	0.0472–0.0599	[22]
Bound wheat straw fibers	130.0	0.0466–0.0569	[22]
Hybrid of Eucalyptus globulus leaves + wheat straw fibers	211.0	0.0460–0.0574	[22]
Hybrid (date palm tree surface fibers + apple of Sodom fibers)	114.0–233.0	0.0423–0.0529	[25]
Date palm surface fibers	176–260	0.0475–0.0697	[26]
Bagasse	70–350	0.0460–0.0550	[43]
Straw bale	50–150	0.0380–0.0670	[43]
Rice husk	154–168	0.0464–0.566	[43]
Corn cob	171–334	0.101	[43]
Jute	26.1	0.0458	[44]
Flax	32.1	0.0429	[44]
Technical hemp	30.2	0.0486	[44]
Coconut fiber	40–90	0.0480–0.0576	[45]
Rock wool	40–200	0.033–0.040	[43]
Expanded polystyrene	15–35	0.031–0.038	[43]
Kenaf	30–180	0.034–0.043	[43]

**Table 7:** Sound absorption coefficient (SAC), density, and noise reduction coefficient (NRC) for bound and hybrid samples.

Natural Fiber Materials	Density, kg/m^3^	Thickness of Board or Fiber, m	Sound Absorption Coefficients				NRC	References
			Frequency, Hz					
			250	500	1000	2000		
PALF (Bo, 2)	329	0.013	0.05	0.07	0.05	0.10	0.05	Current study
SFSF (Bo, 4)	248	0.021	0.07	0.13	0.32	0.52	0.25	Current study
WMS (Bo, 6)	472	0.011	0.05	0.07	0.05	0.09	0.05	Current study
(PALF + SFSF) (Hy, 7)	247	0.021	0.03	0.05	0.11	0.13	0.10	Current study
(PALF + SFSF) (Hy, 8)	299	0.022	0.007	0.05	0.25	0.35	0.15	Current study
(PALF + WMS) (Hy, 9)	478	0.011	0.03	0.06	0.13	0.21	0.10	Current study
(PALF + WMS) (Hy, 10)	256	0.026	0.12	0.21	0.91	0.52	0.45	Current study
Kenaf (light)	50	0.06	0.19	0.33	0.68	0.9	0.55	[28]
Kenaf (dense)	100	0.04	0.18	0.32	0.70	0.94	0.55	[28]
Wood (fibers)	100	0.04	0.40	0.50	0.65	0.91	0.60	[28]
Wood (mineralized)	260	0.03	0.10	0.10	0.20	0.40	0.20	[28]
Coconut	60	0.04/0.06	0.2	0.34	0.67	0.79	0.50	[28]
Cork	100	0.03	0.02	0.10	0.30	0.86	0.30	[28]
Cane (only wooden)	400	0.04	0.06	0.12	0.47	0.43	0.25	[28]
Fleece (100% polyester) fiber	60	0.0035	0.08	0.12	0.19	0.21	0.15	[48]
Queenscord fiber	160	0.0019	0.05	0.14	0.34	0.30	0.20	[48]
Mesh fiber	100	0.0033	0.18	0.02	0.05	0.07	0.10	[48]
Suede fiber	300	0.0006	0.09	0.13	0.24	0.28	0.20	[48]
Wood fiberboard	480	0.018	0.11	0.14	0.21	0.34	0.20	[47]
Wood fiberboard	380	0.025	0.16	0.17	0.31	0.33	0.25	[47]
Wood fiberboard	240	0.018	0.16	0.20	0.30	0.40	0.25	[47]
Banana stem	100	0.010	---	0.05	0.10	0.42	0.15	[49]
Grass	48	0.010	---	0.06	0.15	0.44	0.20	[49]
Palm oil leaves	152	0.010	---	0.05	0.08	0.19	0.10	[49]
Lemongrass	201	0.010	---	0.06	0.15	0.45	0.20	[49]

## Data Availability

Data will be available upon request.

## Nomenclatures

Bo	Bound
DSC	Differential Scanning Calorimetry
DTGA	Differential thermogravimetric analysis
Hy	Hybrid
Lo	Loose
K	Thermal conductivity coefficient
NRC	Noise reduction coefficient
PALF	Pineapple leaf fiber
RH	Relative humidity
SAC	Sound absorption coefficient
SFS	Sunflower seed
SFSF	Sunflower seed fiber
TGA	Thermogravimetric analysis
WMS	Watermelon seed
